# Characterization of Human Genes Modulated by *Porphyromonas gingivalis* Highlights the Ribosome, Hypothalamus, and Cholinergic Neurons

**DOI:** 10.3389/fimmu.2021.646259

**Published:** 2021-06-14

**Authors:** Sejal Patel, Derek Howard, Nityananda Chowdhury, Casey Derieux, Bridgette Wellslager, Özlem Yilmaz, Leon French

**Affiliations:** ^1^ Krembil Centre for Neuroinformatics, Centre for Addiction and Mental Health, Toronto, ON, Canada; ^2^ Department of Oral Health Sciences, Medical University of South Carolina, Charleston, SC, United States; ^3^ Department of Microbiology and Immunology, Medical University of South Carolina, Charleston, SC, United States; ^4^ Campbell Family Mental Health Research Institute, Centre for Addiction and Mental Health, Toronto, ON, Canada; ^5^ Department of Psychiatry, University of Toronto, Toronto, ON, Canada; ^6^ Institute for Medical Science, University of Toronto, Toronto, ON, Canada

**Keywords:** Alzheimer’s disease, cholinergic system, gingipains, transcriptomics, hypothalamus, *Porphyromonas gingivalis* (*P. gingivalis*)

## Abstract

*Porphyromonas gingivalis*, a bacterium associated with periodontal disease, is a suspected cause of Alzheimer’s disease. This bacterium is reliant on gingipain proteases, which cleave host proteins after arginine and lysine residues. To characterize gingipain susceptibility, we performed enrichment analyses of arginine and lysine proportion proteome-wide. Genes differentially expressed in brain samples with detected *P. gingivalis* reads were also examined. Genes from these analyses were tested for functional enrichment and specific neuroanatomical expression patterns. Proteins in the SRP-dependent cotranslational protein targeting to membrane pathway were enriched for these residues and previously associated with periodontal and Alzheimer’s disease. These ribosomal genes are up-regulated in prefrontal cortex samples with detected *P. gingivalis* sequences. Other differentially expressed genes have been previously associated with dementia (*ITM2B*, *MAPT*, *ZNF267*, and *DHX37*). For an anatomical perspective, we characterized the expression of the *P. gingivalis* associated genes in the mouse and human brain. This analysis highlighted the hypothalamus, cholinergic neurons, and the basal forebrain. Our results suggest markers of neural *P. gingivalis* infection and link the cholinergic and gingipain hypotheses of Alzheimer’s disease.

## Introduction

*Porphyromonas gingivalis* (*P. gingivalis*), a keystone species in the development of periodontal disease is believed to play a pathogenic role in several systemic inflammatory diseases (1). This Gram-negative anaerobe is unique in its ability to secrete gingipain proteases, which are its primary virulence factors and are required for its survival *in vivo* (2). *P. gingivalis* is asaccharolytic and uses gingipains to degrade host peptides for nutrition and energy. These gingipain peptidases are cysteine proteases that cleave bonds after arginine (RgpA and RgpB) and lysine (Kgp) (3, 4). These two positively charged amino acid residues facilitate binding of negatively charged nucleic acids (5, 6). This electrostatic relationship suggests gingipains may severely disrupt host cell protein-RNA and protein-DNA interactions.

Positive associations between periodontal disease and orodigestive cancer (7), rheumatoid arthritis (8), heart disease (9), male infertility (10), and Alzheimer’s disease have been reported (11, 12). Several studies have found links that implicate *P. gingivalis* in these associations. For example, serum *P. gingivalis* antibody levels are a risk factor for orodigestive cancer death (13), and are more common in rheumatoid arthritis subjects (14). More directly, *P. gingivalis* expresses enzymes that convert arginine residues to citrulline, which is thought to trigger inflammatory responses in rheumatoid arthritis (15). In the context of heart disease, *P. gingivalis* DNA has been found in human atherosclerotic plaques (16, 17) and gingipains modify high- and low-density lipoproteins (18). More specifically, arginine‐specific gingipains are able to fragment Apolipoprotein E (ApoE) (18). *ApoE* is the strongest genetic risk factor for late-onset Alzheimer’s disease, differences between the genetic variants increase the number of arginine residues at two positions. The lowest risk ApoE2 isoform encodes only cysteines at these positions, while ApoE3 contains one arginine and ApoE4, which confers the highest risk contains two (19). This connection to genetic risk is supported by significant evidence of *P. gingivalis* and specifically gingipains in Alzheimer’s disease pathogenesis (11, 20–23). This evidence linking *P. gingivalis* to several chronic diseases combined with the direct link between arginine count and genetic risk for Alzheimer’s disease motivated our genome- and brain-wide study of gingipain susceptibility.

In this study, we sought to determine which human proteins are susceptible to gingipain cleavage by characterizing proteins with high proportions of arginine and lysine. Motivated by evidence linking *P. gingivalis* to Alzheimer’s disease, we tested if the genes encoding these proteins are differentially expressed in brain tissue with detected *P. gingivalis* RNA. We then performed neuroanatomical enrichment analyses to better understand tissue specific susceptibility. We extend these analyses to a single-cell atlas of the mouse nervous system to identify cell-types that may be specifically susceptible to gingipains.

## Methods

### Amino Acid Distribution Analysis

Translated human protein sequences were obtained from GENCODE version 32 (24). Amino acid proportions were mean averaged across multiple transcripts that were annotated to the same gene symbol. Protein sequences annotated to more than one gene were removed to prevent amplification of single sequences in the following enrichment analyses.

### Gene Ontology Enrichment Analysis

The Gene Ontology (GO) provides gene-level annotations that span specific cellular components, biological processes, and molecular functions (25). These annotations, defined by GO terms, were required to have annotations for 10 to 200 tested genes (6,807 unique GO groups annotating 14,655 unique genes). To test for enrichment, we sorted the genes from the most enriched to the most depleted proportions of arginine and lysine residues. Within this ranking, the area under the receiver operating characteristic curve (AUC) was used to test for gene ontology terms that are enriched in either direction (enriched: AUC > 0.5, depleted: AUC < 0.5). The Mann–Whitney U test was used to determine statistical significance with FDR correction for the GO groups tested. We used GO annotations from the GO.dB and org.Hs.eg.db packages in R, version 3.8.2, which were dated April 24, 2019 (26, 27).

### Gingipain Activity Assay

Gingipain activity was tested against the 70S ribosome of Escherichia coli (New England Biolabs, Ipswich, MA). Recombinant Lys-gingipain (rKgp; CUSABIO, Houston, TX) or recombinant Arg-gingipains (rRgpA and rRgpB; CUSABIO) at 120nM concentrations were incubated with 5 µg of ribosome in TC150 buffer (50 mM Tris, pH 8.0; 150 mM NaCl, 5 mM Cysteine, 5 mM CaCl2) (28) for 1, 3, 6, or 12 hours at 30°C. One unit of recombinant human Caspase-3 (Sigma, St. Louis, MO), a cysteine protease, was used as a control favoring aspartic acid rich peptides to lysine- or arginine-rich peptides (29, 30). All the reactions were ceased *via* boiling with a laemmli sample buffer (Bio-Rad, Hercules, CA) for 5 minutes. The samples were then resolved by SDS PAGE using 4–20% Criterion TGX stain-free gel (Bio-Rad) at 100V for 80 min. Gels were visualized after staining with 0.25% coomassie brilliant blue (in 50% methanol and 7% acetic acid) for 2-4 hours, followed by de-staining (10% methanol and 7% glacial acetic acid) until bands were visible. The assay was also repeated at 37°C and/or using twofold amounts of gingipains (240 nM) for 1 and 3 hours. In all assays, ribosomes without any enzymes or enzymes without any ribosomes were used as reagent controls. To confirm that the gingipains used in this study were biologically active, each of them was tested against recombinant human ApoE4 protein (Novus Biologicals, Centennial, CO), a known substrate of gingipains (18, 20).

### Prefrontal Cortex Differential Expression

Gene expression profiles from a postmortem study of Parkinson’s disease were used to test for gene expression differences in tissue samples with *P. gingivalis* reads (31). To exclude confounding effects of disease processes, we limited our analyses to the 44 neurologically normal control samples of this study. As detailed by Dumitriu et al., prefrontal cortex samples were profiled with Illumina’s HiSeq 2000 sequencers. All samples were from males of European ancestry. Neuropathology reports were used by Dumitriu et al. to exclude brains with Alzheimer’s disease pathology beyond that observed in normal aging.

We used the Sequence Read Archive analysis tool to identify samples with sequencing reads that align to the *P. gingivalis* genome (32). To test for differential expression, we obtained the normalized count matrix for GSE68719 (GSE68719_mlpd_PCG_DESeq2_norm_counts.txt.gz). Batch information was obtained from the Gemma bioinformatics system (33). To identify genes differentially expressed in samples with detected *P. gingivalis*, we modelled log(expression +1) with an ordinary least squares linear model with covariates for age, RIN, PMI, and total bases read. Gene ontology enrichment analyses were performed using the same AUC method described above.

To examine differential expression of cell-type markers, we used the top marker genes obtained from a single cell study of the adult human temporal cortex (34). This study used gene expression profiles to cluster cells into astrocyte, neuron, oligodendrocyte, oligodendrocyte precursor, microglia and endothelial groups. These marker genes were used to calculate AUC values.

### Gene Expression Processing for Anatomical Enrichment Analysis

The Allen Human Brain Atlas was used to determine regional enrichment of the genes identified in the differential expression analyses throughout the brain (35). Thus, in contrast to the differential analyses that focused on samples from the cerebral cortex, this approach used the whole brain, and expression profiles were summarized to named brain regions. For each donor, samples mapping to the same-named brain region were mean-averaged to create a single expression profile for each region. Values from analogous named brain regions of both hemispheres were pooled because no significant differences in molecular architecture have been detected between the left and right hemispheres (36). Expression levels of the 48,170 probes were then summarized by mean averaging for each of 20,778 gene transcripts. Expression values were next converted to ranks within a named brain region, and then z-score normalized across brain regions. For the analyses that combine across donors, z-scores for each donor were averaged to obtain a single gene-by-region reference matrix of expression values.

### Mouse Nervous System Gene Expression Data

The RNA sequencing data that were used to characterize gene expression across the mouse nervous system were obtained from Zeisel et al. (37). This dataset of more than 500,000 isolated cells from 19 defined regions was obtained from male and female mice that ranged in age from 12 to 56 days old. Following quality control, Zeisel et al. identified 265 clusters of cells. These clusters are thought to represent different cell types. Aggregate expression values for the identified transcriptomic cell-type clusters were obtained from the Mousebrain.org downloads portal. These values were log(expression+1) transformed and then z-scored at the gene level. We restricted our analysis to genes with human homologs by filtering mouse gene symbols for those that have human homologs (38). Expression z-scores are then ranked genome-wide within a defined cell-type cluster.

### Neuroanatomical and Cell-Type Cluster Enrichment Analysis

To calculate enrichment for gene sets of interest within a brain region or a cell type, z-scores in the processed expression matrices are first ranked. This provides a genome-wide list for each cell type or brain region with high ranks marking specific and low for depleted expression. We project our genes of interest into these ranked lists to determine enrichment. The AUC statistic was used to quantify if the genes of interest are more specifically expressed (ranked higher) in this sorted list of genes for a specific region or cell cluster. In this context, AUC > 0.5 means the associated genes have specific or enriched expression in a brain region/cell-type cluster. For AUC < 0.5, the reverse is true, with a bias toward lower relative expression. The Mann-Whitney U test was used to test the statistical significance, and the FDR procedure was used to correct for testing of multiple brain regions or cell clusters within a dataset.

## Results

### Genome-Wide Search Reveals a Range of Arginine and Lysine Proportions

The average proportion of arginine or lysine residues was 11.4% across the 20,024 human protein-coding genes in our analysis. A broad range was observed, with 95% of genes having proportions between 5.5% and 19.4% (full listing in **Supplement Table 1**). The gene with the highest proportion was Ribosomal Protein L41 (*RPL41*, 68%) and joined three other ribosomal proteins within the top ten list (*RPL39*, *RPS25*, and *RPL19*). This high proportion of arginine and lysine in these top proteins suggests they are highly susceptible to gingipain cleavage.

### Ribosomal and DNA Packaging Genes Are Enriched for Arginine and Lysine Residues

To characterize the proteins with high arginine and lysine residues genome-wide, we performed a GO enrichment analysis. Of the 6,806 tested GO groups, 881 are enriched for higher proportions of arginine and lysine after multiple test correction (top ten in **Table 1**, full listing in **Supplement Table 2**). In agreement with inspection of the top ten proteins, genes encoding parts of the ribosomal subunit are the most strongly enriched with one in five residues being arginine or lysine on average (AUC = 0.87, p_FDR_ < 10^-58^). The remaining top groups largely contain ribosomal protein genes. Ranked 15th are genes annotated to the nucleosome, which includes many histones (60 genes, 20.9% average proportion, AUC = 0.926, p_FDR_ < 10^-29^). The high enrichment for this set is partially due to 9 highly similar protein sequences from a histone microcluster that has the same arginine and lysine proportion (22.2%). Overall, this enrichment for positively charged arginine and lysine residues mirrors their known ability to facilitate the binding of DNA and ribosomal RNA (6, 39).

**Table 1:** Top GO groups enriched for high arginine + lysine proportion.

Title	ID	Gene count	AUC	p	p_FDR_
ribosomal subunit	GO:0044391	167	0.871	1.78E-61	1.16E-57
structural constituent of ribosome	GO:0003735	114	0.924	3.16E-55	1.03E-51
protein targeting to ER	GO:0045047	97	0.893	6.57E-41	1.43E-37
establishment of protein localization to endoplasmic reticulum	GO:0072599	101	0.881	4.38E-40	7.16E-37
cytosolic ribosome	GO:0022626	93	0.891	5.79E-39	6.79E-36
SRP-dependent cotranslational protein targeting to membrane	GO:0006614	92	0.893	6.23E-39	6.79E-36
large ribosomal subunit	GO:0015934	104	0.869	7.96E-39	7.44E-36
cotranslational protein targeting to membrane	GO:0006613	95	0.879	1.58E-37	1.29E-34
nuclear-transcribed mRNA catabolic process, nonsense-mediated decay	GO:0000184	116	0.842	2.54E-37	1.84E-34
translational initiation	GO:0006413	181	0.762	3.66E-34	2.39E-31

Within the top ten most enriched GO groups, ‘SRP-dependent cotranslational protein targeting to membrane’ appears to be the most specific with the lowest number of annotated genes (**Figure 2B**). These genes are primarily components of the cytosolic ribosome that facilitate translation into the endoplasmic reticulum (ER) but also include genes encoding the signal recognition particle (SRP) and its receptor. For brevity, we refer to this GO group as the ‘ER translocation’ genes. This specific GO group has been previously associated with disorders that *P. gingivalis* is believed to play a pathogenic role. Specifically, a spatial transcriptomics study reported higher expression of the ER translocation genes in inflamed areas of periodontitis-affected gingival connective tissue compared to non-inflamed areas (40). A second study that examined peri-implant soft tissue found that ER translocation genes are expressed at higher levels in diseased mucosa samples (41). In the context of Alzheimer’s disease, the ER translocation genes are upregulated across neuroinflammation, in cases from a Caribbean Hispanic postmortem study, amyloid associated dystrophic microglia, and in regions of Alzheimer’s disease-associated hypometabolism (42–44). Given this upregulation in periodontal and Alzheimer’s disease, we pursued further characterization of the ER translocation genes.

**Figure 1:**
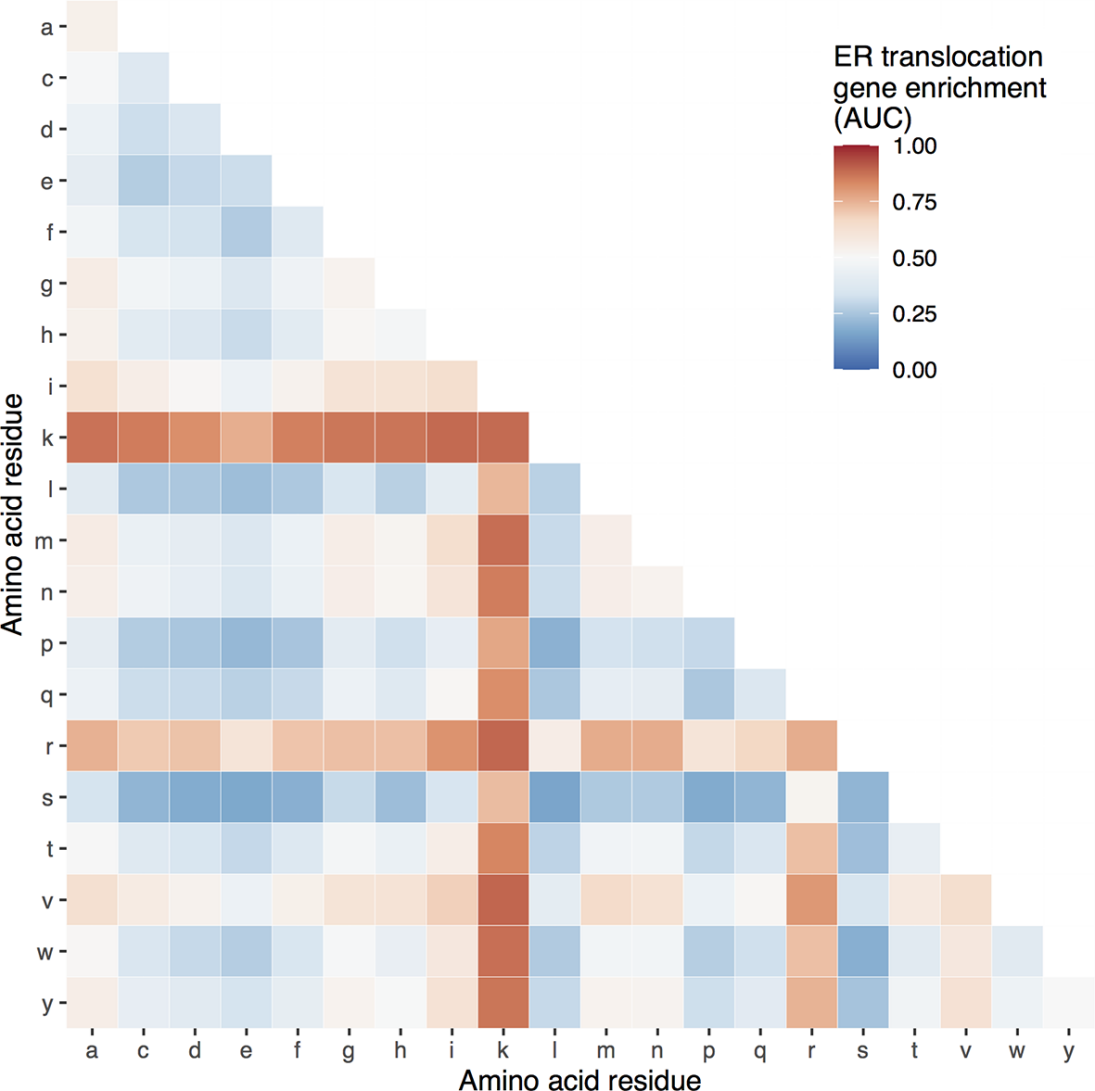
Heatmap of ER translocation gene enrichment for proportions of different amino acid pairs. Relative to all other genes, proportions of specific amino acid pairs for the ER translocation genes range from high (red) to low (blue).

Genome-wide, the ER translocation genes have a high proportion of arginine and lysine, but it’s not clear if other residues are enriched. To determine the specificity of this enrichment, we tested proportions of other amino acids. For single residues, lysine (AUC = 0.89) and arginine (AUC = 0.76) are the most enriched in the ER translocation genes, followed by valine and isoleucine (AUC = 0.64 and 0.63, respectively) (**Supplement Table 3**). For pairs, **Figure 1** shows the AUC values of the 190 possible amino acid combinations. Only the lysine and valine proportion have a higher enrichment score, which is slightly higher (AUC = 0.896 compared to 0.894 for arginine + lysine). Still, this combination is less frequent (18.3% of residues compared to 20.4%). Overall, the ER translocation genes are strongly and specifically enriched for arginine and lysine residues, suggesting they are particularly susceptible to cleavage by gingipains.

### Gingipains Showed Very Low Activity Against 70S Ribosomes

Recombinant lysine and arginine gingipains exhibited weak to no activity against the *E. coli* 70S ribosome at 30°C for a 1-3 hour incubation period (**Supplement Figure 1**). Cleavage activity did not change upon increases in gingipains’ concentration, incubation temperature (37°C), or duration (data not shown). Similarly, Caspase-3, which was used as a control cysteine protease did not demonstrate any detectable activity (cleavage) of the ribosome. The recombinant gingipains used were biochemically active as confirmed by the cleavage of recombinant human ApoE4 protein in control assays.

### Hundreds of Genes Are Differentially Expressed In Brain Tissue With *P. gingivalis* Reads

Guided by the findings of the upregulation of ER translocation genes in the context of Alzheimer’s disease, we tested for direct associations with *P. gingivalis* in postmortem brain tissue. We examined control samples that lacked any neurodegenerative disease pathology to remove any late-stage signals of Parkinson’s or Alzheimer’s disease. In this all-male dataset, age ranged from 46 to 97 years old. Within the 44 prefrontal cortex samples, *P. gingivalis* sequencing reads were detected in ten. In contrast, reads from the two other bacteria in the red complex, which is associated with severe periodontal disease, were not detected (*Tannerella forsythia* and *Treponema denticola*). There was no difference between age, RNA integrity number, and postmortem interval between the samples with and without detected *P. gingivalis* reads (all p > 0.33). In contrast, there was a significant difference in the number of bases sequenced, with more reads in the samples with detected *P. gingivalis*. All four of these variables were covariates in our differential expression model. Importantly, batch information is confounded with the detected *P. gingivalis* reads. Specifically, all 10 ten samples with detected *P. gingivalis* reads were sequenced on one of the three sequencing machines used for the study (device DGL9ZZQ1). This adds uncertainty to our results from this dataset. We also tested for differential expression in samples from this specific device but note this reduces the sample size to 14 from 44 for this specific analysis.

**Figure 2:**
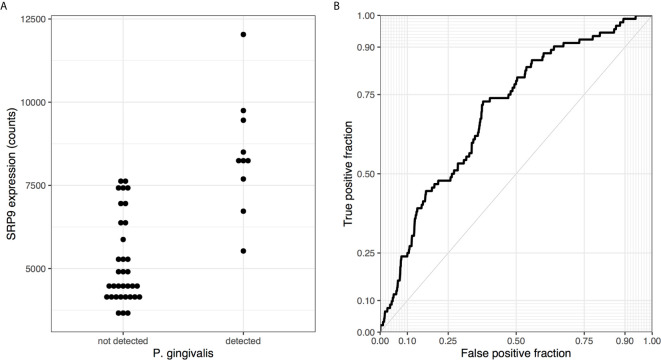
Visualization of differential expression results. **(A)** Dotplot of *SRP9* expression is plotted for samples with and without *P. gingivalis* reads. **(B)** ROC for the ER translocation genes when all genes are ranked according to direction and significance [signed log(p-value)].

In total, 2,189 of the 15,936 tested genes were differentially expressed. More genes were down- than up-regulated in samples with detected *P. gingivalis* reads (1247 versus 942). Arginine and lysine proportion was different between these two sets of genes with an average proportion of 12.8% for the up-regulated genes versus 11.4% for the down-regulated genes (p < 10^-16^). The top ten most up- and down-regulated genes are provided in **Table 2** (full listing in **Supplement Table 4**). The third most up-regulated gene is signal recognition particle 9 (*SRP9*, p_FDR_ < 0.0013, **Figure 2A**). *SRP9* is a member of the ER translocation gene set that forms a heterodimer with the next ranked SRP gene (*SRP14*, p_FDR_ < 0.02) (45). The strongest downregulated genes include putative RNA helicases (*DHX30*, *DHX37*) and other genes involved in the regulation of global gene transcription (*ZNF696*). No genes survive multiple test correction when differential expression is tested in the smaller set samples profiled by device DGL9ZZQ1. However, *ZNF696* and *DHX37* remain down-regulated in these samples (uncorrected p < 0.05).

**Table 2:** Top ten most up- and down-regulated differentially expressed genes in the prefrontal cortex with detected *P. gingivalis* reads.

Name	Symbol	Estimate	p_FDR_
integral membrane protein 2B	*ITM2B*	0.446	0.00107
transmembrane and coiled-coil domains 1	*TMCO1*	0.357	0.00129
signal recognition particle 9	*SRP9*	0.556	0.00129
eukaryotic translation initiation factor 3 subunit M	*EIF3M*	0.273	0.00129
RAB28, member RAS oncogene family	*RAB28*	0.463	0.00133
anti-silencing function 1A histone chaperone	*ASF1A*	0.411	0.00134
inner mitochondrial membrane peptidase subunit 1	*IMMP1L*	0.555	0.00134
MNAT1 component of CDK activating kinase	*MNAT1*	0.389	0.0014
zinc finger protein 267	*ZNF267*	0.754	0.00159
mitochondrial calcium uptake 2	*MICU2*	0.393	0.00165
	….		
transmembrane protein 63B	*TMEM63B*	-0.417	0.000998
ubiquitin like modifier activating enzyme 1	*UBA1*	-0.372	0.000998
dynein cytoplasmic 1 heavy chain 1	*DYNC1H1*	-0.446	0.000998
trafficking protein particle complex 9	*TRAPPC9*	-0.406	0.000963
ATP binding cassette subfamily F member 3	*ABCF3*	-0.362	0.000963
fatty acid synthase	*FASN*	-0.701	0.000812
transmembrane protein 8B	*TMEM8B*	-0.4	0.000812
DEAH-box helicase 37	*DHX37*	-0.513	0.000745
zinc finger protein 696	*ZNF696*	-0.311	0.000745
DExH-box helicase 30	*DHX30*	-0.4	0.000745

### ER Translocation Genes Are Enriched for Upregulation in Samples With*P. gingivalis* Reads

To summarize the hundreds of differentially expressed genes, we again used the AUC metric. Genes were ranked from the most up- to down-regulated in the samples with detected *P. gingivalis*. Of the 6,538 tested GO groups, 23 were significantly up-regulated, and 49 were down-regulated after multiple test correction (full listing in **Supplement Table 5**). Across this ranking, ER translocation genes were strongly up-regulated and is the fourth most significant GO group (AUC = 0.698, p_FDR_ < 10^-7^). Similar GO groups that primarily contain genes encoding ribosomal proteins make up the other top ten up-regulated groups. Of these ten, all are in the top ten list of groups that are enriched for arginine and lysine residues except the ‘protein localization to endoplasmic reticulum’ and ‘protein targeting to membrane’ groups. Within the top ten most down-regulated GO groups, ‘homophilic cell adhesion *via* plasma membrane adhesion molecules’ ranked first, followed by several synapse associated groups and ‘ATP biosynthetic process’ (all p_FDR_ < 0.005). In the context of amino acid residues, we note that genes annotated to ‘aminoacyl-tRNA ligase activity’ are also down-regulated (AUC = 0.33, p_FDR_ < 0.05). In summary, samples with detected *P. gingivalis* reads have higher expression of ER translocation genes.

Given the down-regulation of synapse genes, we next tested if cell-type specific markers are differentially expressed. Of the six cell-types, only genes marking endothelial cells (AUC = 0.805, p_FDR_ < 0.00005) and astrocytes (AUC = 0.682, p_FDR_ < 0.02) were enriched, with higher expression in samples with *P. gingivalis* reads. In contrast, markers of oligodendrocyte precursors, oligodendrocytes, neurons, and microglia were not enriched. Specifically, the 20 neuronal markers are equally split between up- and down-regulated with no genes reaching statistical significance at an alpha of 0.05 (uncorrected). In summary, differential expression of cell-type markers suggests changes in endothelial and astrocyte cell states or proportions but not neurons.

### Genes Up-Regulated in Samples With *P. gingivalis* Reads Are Highly Expressed in the Anterior Hypothalamic Area

To further characterize the genes associated with *P. gingivalis*, we determined which brain regions are enriched for their expression. While these genes were identified in samples from the prefrontal cortex, it is believed neocortical degeneration doesn’t occur until later stages of Alzheimer’s disease (46). Testing for neuroanatomical enrichment may reveal other regions of interest. In this analysis, for a given brain region, the genome was ranked from the most specifically expressed gene to the most depleted gene (relative to the rest of the brain). Similar to the preceding analyses, we use the AUC metric to test if genes of interest rank higher in this list to determine region-specific expression. In total, 82 of 232 tested brain regions are enriched for high expression of the genes up-regulated in samples with detected *P. gingivalis* (**Supplement Table 6**). The anterior hypothalamic area most specifically expresses these genes (AUC = 0.740, p_FDR_ < 10^-120^). This result is consistent when tested in individual brain hemispheres (n = 2 brains, left AUC: 0.738; right: 0.698). Within the top ten most enriched regions, two other hypothalamic regions appear (**Table 3**, AUCs > 0.68, both p_FDR_ < 10^-76^). Notably, most of the top regions border ventricles (medial habenular nucleus, thalamic paraventricular nuclei, substantia innominata, central gray of the pons, paraventricular nucleus of the hypothalamus, and septal nuclei).

**Table 3:** Top ten regions enriched for higher expression of genes up-regulated in samples with detected *P. gingivalis* reads.

Region name	AUC	p	p_FDR_
anterior hypothalamic area	0.740	1.16E-135	2.68E-133
medial habenular nucleus	0.702	2.96E-97	3.44E-95
paraventricular nucleus of the hypothalamus	0.684	4.60E-81	3.56E-79
lateral hypothalamic area, anterior region	0.683	1.85E-79	1.08E-77
septal nuclei	0.682	6.28E-79	2.92E-77
substantia innominata	0.679	8.62E-77	3.33E-75
central gray of the pons	0.675	3.77E-73	1.25E-71
midbrain reticular formation	0.672	2.03E-70	5.88E-69
paraventricular nuclei, right of thalamus	0.658	1.00E-59	2.59E-58
paraventricular nuclei, left of thalamus	0.656	1.26E-58	2.92E-57

### Cells in the Mouse Peripheral Nervous System and Cholinergic Neurons Highly Express Genes Up-Regulated in Samples With *P. gingivalis* Reads

We next characterized the expression patterns of the genes up-regulated in samples with detected *P. gingivalis* at a finer resolution in the mouse nervous system (37). Similar to the regional analyses above, we ranked each gene from the most specific to depleted expression for each transcriptomic cell type cluster. The 873 mouse homologs of the up-regulated genes are enriched for specific expression in 79 of the 265 tested transcriptomic cell type clusters (**Supplement Table 7**). The top two enriched clusters are nitrergic enteric neurons (both AUC > 0.67, p_FDR_ < 10^-65^). The next three most enriched are cholinergic neurons with probable locations listed as the sympathetic ganglion or myenteric plexus of the small intestine (all AUC > 0.66, p_FDR_ < 10^-56^). The top 19 most enriched clusters are located in the peripheral nervous system. Focusing on the central nervous system, the top two clusters described as “Afferent nuclei of cranial nerves VI-XII” and “Cholinergic neurons, septal nucleus, Meynert [sic] and diagonal band” (both AUC < 0.59, p_FDR_ < 10^-19^). Broadly, cells in the mouse peripheral nervous system and cholinergic neurons strongly express genes that are up-regulated in samples with detected *P. gingivalis.*

### ER Translocation Genes Are Highly Expressed in the Substantia Innominata

**Figure 3:**
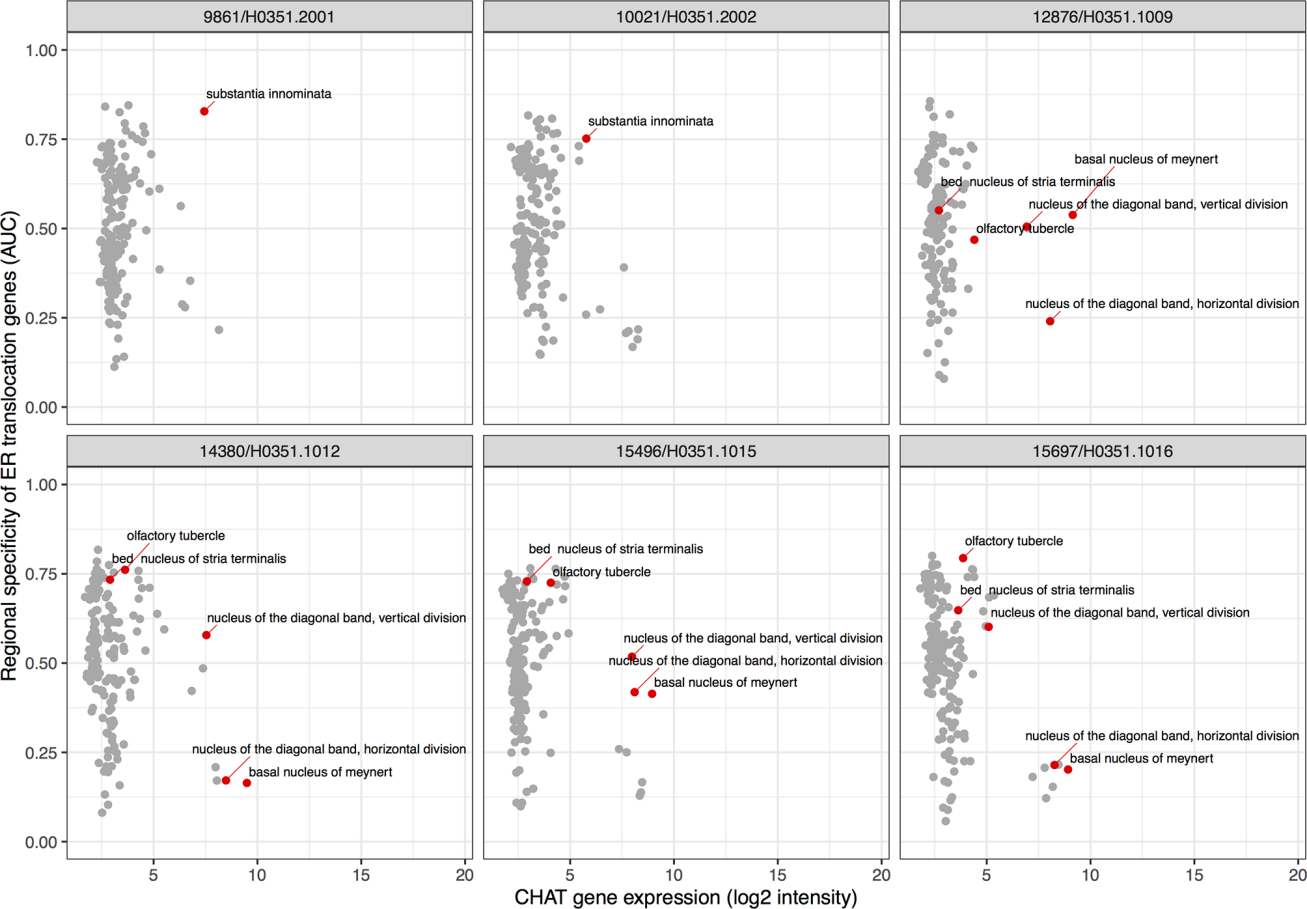
Scatter plots showing specific enrichment for ER translocation genes (y-axis) and Choline O-Acetyltransferase (*CHAT*) gene expression on the x-axis in each brain. Each point is a brain region with red marking the substantia innominata and its nuclei.

To extend our results beyond protein level susceptibility, we next identified brain regions that express high levels of the ER translocation genes. This provided a neuroanatomical perspective of arginine and lysine proportions. While our preceding analyses used genes identified from brain tissue samples, the ER translocation genes were identified from analyses of protein sequences alone. This provides independence from tissue- and state-specific expression. In a combined analysis of all six brains, 83 of the 232 brain regions showed overexpression of the ER translocation genes. The substantia innominata was top-ranked with the most specific expression of the ER translocation genes (AUC = 0.846, p_FDR_ < 10^-28^, full listing in **Supplement Table 8**). This enrichment appears when hemispheres are tested separately (n = 2 brains, AUC left: 0.847; right: 0.816). Of the nine remaining top 10 regions, seven are located near the substantia innominata: internal and external globus pallidus, substantia nigra pars reticulata, the septal nuclei, nucleus accumbens, subcallosal cingulate gyrus, head of the caudate nucleus (AUCs > 0.774, all p_FDR_ < 10^-18^). Of the 34 assayed cerebellar cortex regions, all are significantly enriched for expression of the ER translocation genes. Within specific brains, the substantia innominata is the 10th ranked brain region (of 194) for selective expression of ER translocation associated genes in donor 10021/H0351.2002, and is ranked 3rd of 182 in donor 9861/H0351.2001. There are no samples from the substantia innominata in the other four donors, but its constituent nuclei are. Testing for anatomical enrichment of the ER translocation associated genes in these nuclei reveals a highly heterogeneous pattern (**Figure 3**). A key characteristic of the substantia innominata is a high proportion of cholinergic neurons (47). Alzheimer’s disease has been previously associated with cholinergic neuron loss in the basal forebrain and deficits in choline O-acetyltransferase (encoded by *CHAT*) (48–50). **Figure 3** shows the relationships between *CHAT* gene expression and ER translocation genes across the brain, marking the substantia innominata as having high expression of both *CHAT* and the ER translocation genes.

### ER Translocation Genes Are Highly Expressed in Mouse Hypothalamic and Cholinergic Neurons

Mirroring our analyses of *P. gingivalis* associated genes, we next characterized the expression patterns of the ER translocation genes in the mouse nervous system. We observe that the 79 mouse homologs of the ER translocation genes are not evenly expressed across clusters in this single-cell atlas. The top 20 most enriched transcriptomic cell type clusters are provided in **Table 4** (full listing in **Supplement Table 9**). Four cholinergic enteric neuron clusters are within the top ten cell clusters. Of the 14 cholinergic clusters in this atlas, 12 are enriched for ER translocation gene expression (all AUC > 0.5 and p_FDR_ < 0.05). Relative to the 265 tested cell type clusters, the cholinergic groups are enriched for higher AUC values (p < 0.0001, Mann-Whitney U test). The top-ranked cholinergic cell clusters from brain tissue are listed as telencephalon interneurons with an annotated location of striatum and amygdala (TECHO, AUC = 0.822, ranked 21st). In **Table 4**, it is also clear that hypothalamic cells strongly express the ER translocation genes. Of the 14 total hypothalamic cell clusters, 7 are enriched for ER translocation gene expression (AUC > 0.57, p_FDR_ < 0.05). All of the hypothalamic cell type clusters enriched for ER translocation genes are peptidergic (or produce peptide hormone precursors). Three of the top hypothalamic transcriptomic cell types were annotated with locations that are in or near the basal forebrain. Specifically, the HYPEP5 cluster lists the nucleus of the diagonal band and the HYPEP8 cluster names the medial septal nucleus as probable locations of those clustered cells (37). While substantia innominata is not mentioned in this atlas, both the mouse and human regional analyses highlight the basal forebrain, hypothalamus and cholinergic system.

**Table 4:** Top 20 transcriptomic cell type clusters enriched for higher expression of the ER translocation genes.

Cluster_ID	Name	AUC	P_FDR_
ENT5	Cholinergic enteric neurons	0.917	3.68e-36
HYPEP5	Vasopressin-producing cells, hypothalamus	0.906	1.38e-34
HYPEP4	Oxytocin-producing cells, hypothalamus	0.882	7.67e-31
ENT6	Cholinergic enteric neurons	0.879	1.94e-30
ENT3	Nitrergic enteric neurons	0.876	5.93e-30
ENT8	Cholinergic enteric neurons, VGLUT2	0.876	6.26e-30
HYPEP8	Peptidergic neurons, hypothalamus	0.862	7.13e-28
HBSER3	Serotonergic neurons, hindbrain	0.858	2.37e-27
ENT4	Cholinergic enteric neurons	0.858	2.84e-27
DGNBL2	Granule neuroblasts, dentate gyrus	0.857	3.14e-27
MEGLU14	Glutamatergic projection neurons of the raphe nucleus	0.851	2.81e-26
ENMFB	Enteric mesothelial fibroblasts	0.849	4.27e-26
HYPEP7	Pmch neurons, hypothalamus	0.849	4.87e-26
PER2	Pericytes, possibly mixed with VENC	0.836	3.37e-24
ENT2	Nitrergic enteric neurons	0.836	3.37e-24
HBSER1	Serotonergic neurons, hindbrain	0.835	4.13e-24
ENTG5	Enteric glia	0.833	8.21e-24
VECC	Vascular endothelial cells, capillary	0.832	8.49e-24
ABC	Vascular leptomeningeal cells	0.826	5.41e-23
ENTG4	Enteric glia	0.825	8.41e-23

### High Neuroanatomical Convergence of *P. gingivalis* Associated Genes

**Figure 4:**
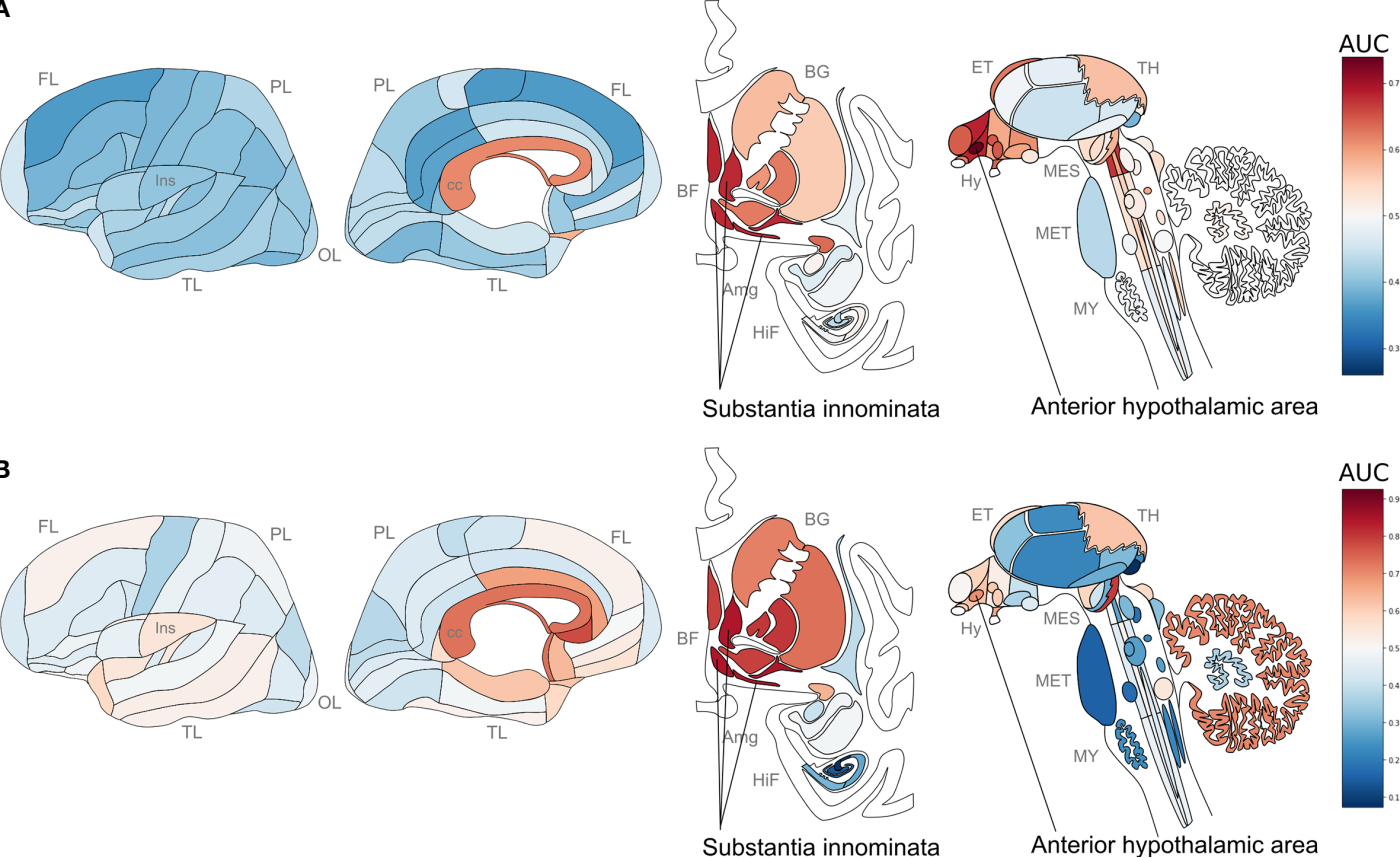
Neuroanatomical heatmaps marking specific expression of the genes up-regulated in samples with *P. gingivalis* reads **(A)** and ER translocation genes **(B)**. AUC values range from depleted expression in dark blue to enriched in dark red. Brain region abbreviations: FL, frontal lobe; PL, parietal lobe; TL, temporal lobe; OL, occipital lobe; BF, basal forebrain; BG, basal ganglia; AmG, amygdala; HiF, hippocampal formation; EP, epithalamus; TH, thalamus; Hy, hypothalamus; MES, mesencephalon; MET, metencephalon; and MY, myelencephalon. Anatomical template images are from the Allen Human Brain Reference Atlas (51).

**Figure 5:**
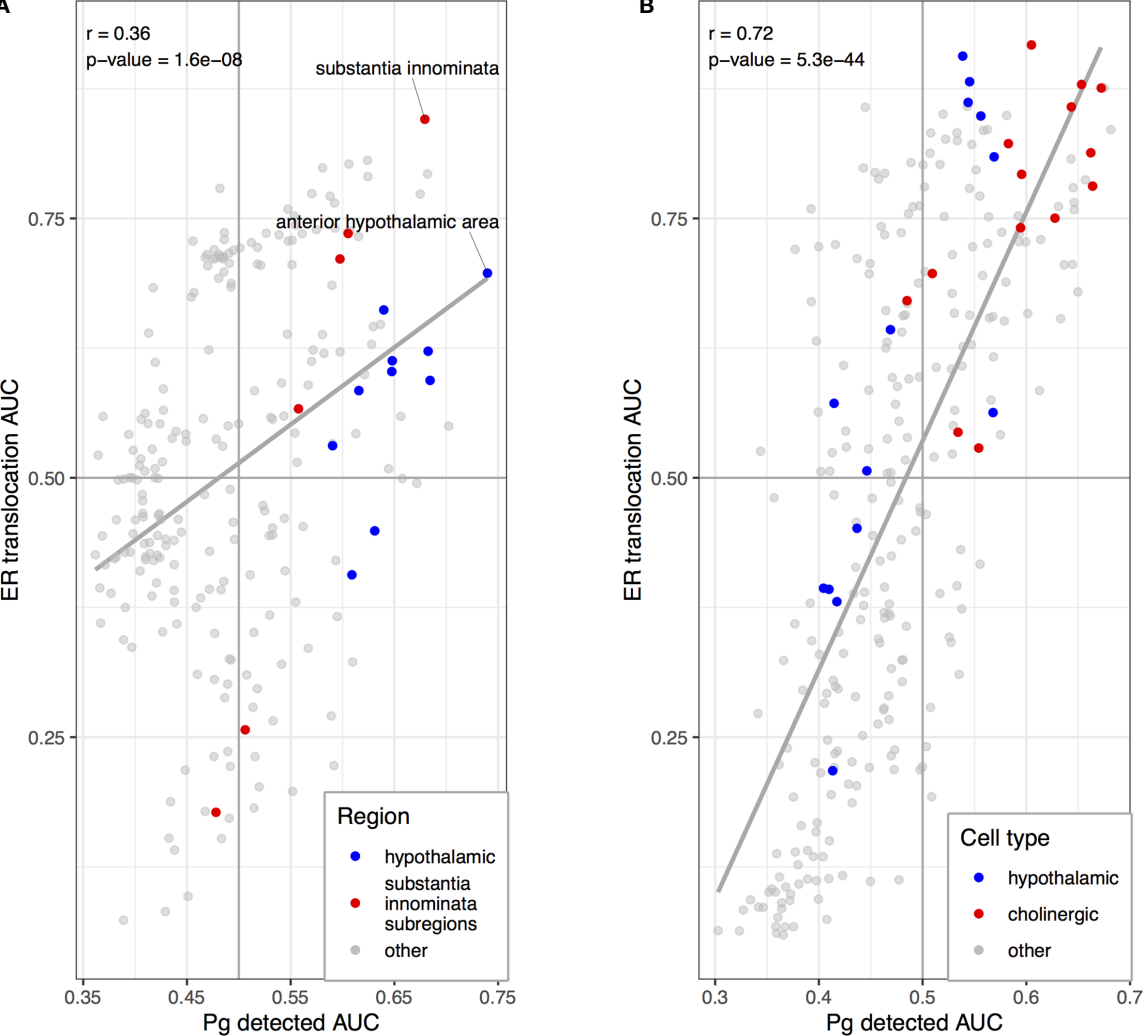
Scatterplots comparing anatomical enrichment of ER translocation genes (y-axis) and up-regulated in samples with *P. gingivalis* reads (x-axis). AUC values are plotted to show enrichment in the human brain atlas **(A)** and the mouse nervous system **(B)**. Hypothalamic regions and transcriptomic cell type clusters are marked in blue. Cholinergic clusters, substantia innominata and its subregions are marked in red. Fitted linear regression lines for all points are shown in grey.

We next tested for overlap between the two brain-wide patterns derived from separate sources of *P. gingivalis* associated genes. Within the top ten regions, the central gray of the pons, septal nuclei, and substantia innominata appear in both enrichment results. More specifically, the anterior hypothalamic area, the most enriched region for the genes associated with *P. gingivalis* reads, is the top-ranked hypothalamic region for the ER translocation results (ranked 51 of 232 overall, AUC = 0.697, p_FDR_ < 10^-10^). Conversely, the substantia innominata, which is most enriched for the ER translocation genes, is ranked 4th for genes associated with *P. gingivalis* reads. This spatial convergence within the top hits extends brain-wide with 48 brain regions overlapping between two lists of significantly enriched structures (hypergeometric test, p < 10^-6^) and correlation coefficient of AUC values of 0.36 (p < 10^-7^). This brain-wide spatial agreement is visualized in **Figures 4** and **5A**. As shown in **Figure 5B**, high agreement is also observed in the single-cell atlas of the mouse nervous system (r = 0.72, p < 10^-43^). In particular, enriched expression in cholinergic neurons is evident for both sets of *P. gingivalis* associated genes. In summary, genes associated with gingipain susceptibility and *P. gingivalis* presence are highly expressed in hypothalamic, cholinergic neurons, and basal forebrain regions.

## Discussion

In this study, we characterized protein susceptibility to gingipain cleavage and genes differentially expressed in brain tissue with *P. gingivalis*. As expected, we found that genes with high arginine and lysine proportions are enriched in proteins that bind RNA and DNA. We focused on a specific set of these genes that participate in ER translocation and have previously been shown to be up-regulated in periodontitis, dystrophic microglia, and Alzheimer’s disease (40, 42–44). We directly link these findings to *P. gingivalis* by showing that these genes are also up-regulated in brain tissue with detected *P. gingivalis* RNA. This convergence between proteins susceptible to gingipain cleavage and the transcriptomic response to *P. gingivalis* motivated our neuroanatomical characterization of these genes. In this spatial analysis, we again observe agreement, with enrichment for cholinergic neurons, basal forebrain and hypothalamic regions. Regions near ventricles and peripheral neurons are also enriched, suggesting relevance to *P. gingivalis* brain entry. While gingipain levels have been shown to correlate with tau and amyloid pathology (20–22), we are the first to associate this virulence factor with the cholinergic hypothesis of Alzheimer’s disease.

In our postmortem brain tissue analyses, several differentially expressed genes are related to amyloid processing and inflammation. For example, *ZNF267*, the 9th most up-regulated gene, was selected as one of the ten genes used in a blood-based transcriptomic panel for diagnosis AD (52). The most significantly up-regulated gene, integral membrane protein 2B (*ITM2B*, p_FDR_ < 0.0011), regulates the processing of amyloid-beta and inhibits amyloid aggregation (53). Mutations in this gene that encodes the BRI2 protein cause familial British and Danish dementia (54, 55). We also note that the 12th most downregulated gene encodes Myc associated zinc finger protein (*MAZ*) and is also known as serum amyloid A-activating factor-1 (*SAF-1*), due to its role in regulating serum amyloid A in response to inflammation (56). Expression of the mouse homolog of *MAZ* is increased in an Alzheimer’s disease mouse model (57). Furthermore, murine overexpression increases the risk of severe arthritis (58). Like *P. gingivalis*, serum amyloid A is suspected to be involved in the pathogenesis of arthritis, atherosclerosis, amyloidosis and Alzheimer’s disease (59–62). Lastly, the 3rd most down-regulated gene, *DHX37*, harbors a rare frameshift mutation that segregates with Alzheimer’s disease in one family (63). While our differential expression analysis highlighted ER translocation genes, several others link amyloid processing and diseases associated with *P. gingivalis*. While these differential expression results are uncertain due to a batch confound, the prior associations of these genes suggest follow-up experiments.

Recently, an *in situ* examination of human periodontitis gingiva samples identified a markedly increased presence of microvasculature with heavily invaded microvessels by live *P. gingivalis* (64). In support, we observe strong up-regulation of endothelial marker genes in brain samples with detected *P. gingivalis* reads. This upregulation indicates a higher proportion of endothelial cells. Together, these findings suggest the vasculature as a potential dissemination route for the microorganism from the oral cavity.

Genes that function in cell-cell contact were down-regulated in brain tissue with detected *P. gingivalis*. Specifically, the top two GO groups enriched for down-regulation were “homophilic cell adhesion *via* plasma membrane adhesion molecules” and “regulation of synaptic plasticity”. These are independent signals as only two genes are in both of these sets. A study of cell-free mRNA found that “homophilic cell adhesion *via* plasma membrane adhesion molecules” was the most significantly enriched GO group within genes down-regulated in AD cases (65). In mice, Huang and colleagues found that *P. gingivalis* infection led to synaptic loss in cerebral cortex neurons. They determined this synaptic failure was driven by Cathepsin B mediated interleukin-1β upregulation in leptomeningeal cells (66). In support, a recent study by Haditsch et al. found that neurons derived from human inducible pluripotent stem cells, when infected with *P. gingivalis*, had a significant loss of synapse density (67). This loss was more pronounced than neuron cell death. In agreement, our results do not suggest differences in estimated neuron proportions. It is known that synapse loss is an early and significant event in Alzheimer’s disease (68). In addition, in the aging and Alzheimer’s brain, decreases in synapse number and gene expression are observed but not neuron counts (69, 70). Haditsch and colleagues suggest synapse loss is due to microtubule destabilization caused by tau degradation by gingipains. In support, the most differentially expressed gene in the cell adhesion GO group is microtubule-associated protein tau (MAPT, p_FDR_ < 0.005, 198th most down-regulated gene). However, our results suggest that transcriptional responses may partially cause reduced synapse density and cell adhesion. Cell-to-cell contact has been shown to increase *P. gingivalis* transmission rate (71, 72), suggesting that these transcriptional responses may reduce *P. gingivalis* persistence.

The ribosome, RNA processing, and protein synthesis have been previously associated with mild cognitive impairment, atherosclerosis, and Alzheimer’s disease (73–76). In agreement with gingipain susceptibility, a proteomic study found a lower abundance of RNA splicing proteins in cerebral atherosclerosis (77). Dysregulated splicing is also demonstrated by increased intron retention in Alzheimer’s disease (78). Similarly, RNA quality, which is measured from ribosomal RNA is lower in brain tissue from dementia cases (79). However, gingipains did not clearly cleave the ribosome in our experiments. We suspect this is due to the stability of the ribosome and inability to detect the cleavage of small ribosomal proteins. Pathological tau, a key marker of Alzheimer’s disease, has been shown to affect translational selectivity and associate with ribosomes on the ER [reviewed in (80)]. Linking arginine, tau was also found to strongly bind transfer RNAs with a preference for tRNA^Arg^ (81). Also, major histone acetylation differences have been associated with tau pathology (82). These findings of disruptions in processes involving nucleic acid interactions, match the functions of proteins enriched for arginine and lysine residues.

While proteins that interact with RNA and DNA are broadly enriched for arginine and lysine, protein targeting to the ER is the top GO group enriched for up-regulation in samples with detected *P. gingivalis*. While speculative, the ER translocation genes point to mechanisms that support the gingipain hypothesis of Alzheimer’s disease. The gingipain hypothesis proposes that *P. gingivalis* infection of brain tissue causes Alzheimer’s disease (20). In infected human cells, *P. gingivalis* is found in vacuoles that contain undegraded ribosomes (83). *P. gingivalis* uses amino acids as an energy source (84). Colocalized ribosomes may provide a particularly digestible source of amino acids because of their enrichment for the positively charged residues that gingipains cleave. Mirroring the movement of the SRP after recognizing a signal peptide, cytosolically free *P. gingivalis* colocalizes with the rough ER upon cell entry (85). It then forms autophagosome-like vacuoles, which support intracellular persistence and multiplication (85). It is tempting to speculate that sequestration of ribosomes in autophagosome-like vacuoles by *P. gingivalis* may cause increased transcription of cytosolic ribosomal protein genes because ribosome biogenesis is highly regulated (86).

The anterior hypothalamic area, oxytocin-, orexin- and vasopressin-expressing neurons were strongly enriched for our *P. gingivalis* associated genes. The hypothalamus releases these neuropeptides and peptide hormones after extensive pre-processing in the ER. Evidence of hypothalamic dysfunction and decreases in hypothalamic volume have been found in Alzheimer’s disease patients [reviewed in (87)]. Functionally, significant deficits in sleep and circadian rhythm have been reported with associations to vasopressin in human studies (88). Orexin, which plays a major role in the sleep-wake cycle, has been associated with amyloid pathology in mouse models (89). In Alzheimer’s patients, orexin levels in cerebrospinal fluid were correlated with amyloid-β42, sleep disruption and fragmentation (90). In addition, galanin, which is strongly expressed in the hypothalamic neurons enriched in our analysis, is associated with sleep fragmentation in Alzheimer’s disease (91). Hypothalamic dysfunction in Alzheimer’s disease and its enrichment for *P. gingivalis* associated genes suggest this region is relevant to the gingipain hypothesis.

Our findings associate the substantia innominata and cholinergic neurons with *P. gingivalis* transcriptomic response and gingipain susceptibility. Enrichment in the medial habenula, a region with high expression of nicotinic cholinergic receptors, is also observed (92). While basal forebrain neurons receive inputs from many cells, rodent studies have found 2-11% are from the hypothalamus (93, 94). These circuits that connect regions with the highest *P. gingivalis* associated enrichment have been explored in the context of cognitive impairment and Alzheimer’s disease. Specifically, studies have highlighted the hypothalamic-pituitary-adrenal axis and upstream modulation of specific cortical function (95, 96). More directly, extensive loss of cholinergic neurons in the substantia innominata has been found in Alzheimer’s patients (97). This finding and many others form the basis of the cholinergic hypothesis of AD, which proposes that degeneration of cholinergic neurons in the basal forebrain substantially contributes to cognitive decline in AD patients (49). This subcortical degeneration is thought to occur early in the disease process (98). In the context of the gingipain hypothesis, we note that reduced basal forebrain volume and cholinergic function follow the removal of teeth in rodents (99–101), and oral acetylcholine levels are correlated with periodontal disease severity (102). High expression of the ER translocation genes in cholinergic neurons may be required to support acetylcholinesterase processing in the ER (103). In the mouse atlas, cholinergic neurons in the enteric nervous system had the highest expression of the ER translocation genes. In the context of AD, loss of enteric cholinergic neurons has been observed in a transgenic mouse model of the disease (104). Taken together, our *P. gingivalis* associated genes highlight cholinergic neurons and the substantia innominata, providing an anatomical between the gingipain and cholinergic hypotheses.

## Conclusions

In conclusion, we show that proteins enriched for arginine and lysine residues, which are potential gingipain cleavage sites, bind RNA and DNA. Neuroanatomical analysis of the *P. gingivalis* associated genes marked cholinergic neurons, the basal forebrain, and hypothalamic regions. These results link the gingipain and cholinergic hypotheses of AD. Our findings detail *P. gingivalis* response and susceptibility at the molecular and anatomical levels that suggest new associations relevant to AD pathogenesis.

## Data Availability Statement

Scripts, supplementary tables, and data files for reproducing the analyses are publicly available online at https://figshare.com/articles/dataset/Susceptibility_to_gingipains_and_transcriptomic_response_to_P_gingivalis_highlights_the_ribosome_hypothalamus_and_cholinergic_neurons/12782576 and https://github.com/leonfrench/gingipain_release.

## Author Contributions

SP, DH, and LF designed and performed bioinformatic analyses. NC, CD, BW, and ÖY designed and performed the protease digestion experiments. All authors contributed to the drafting of the manuscript. ÖY and LF supervised the research. All authors contributed to the article and approved the submitted version.

## Funding

This study was supported by the CAMH Foundation, CAMH Discovery Fund, and a National Science and Engineering Research Council of Canada (NSERC) Discovery Grant to LF. This work was also supported by funding from the NIDCR grants, R56DE016593, R01DE030313, and R01DE030313S1.

## Conflict of Interest

LF owns shares in, and has received consulting fees from Cortexyme Inc., a company that is developing gingipain inhibitors to treat neurodegenerative diseases.

The remaining authors declare that the research was conducted in the absence of any commercial or financial relationships that could be construed as a potential conflict of interest.

## Supplementary Material

Supplementary Table 1 Proteome-wide arginine and lysine counts and proportion.

Supplementary Table 2 Gene Ontology enrichment results for arginine and lysine proportions.

Supplementary Table 3 SRP-dependent cotranslational protein targeting to membrane amino acid specificity tests.

Supplementary Table 4 Results of differential expression tests for *P. gingivalis* detection.

Supplementary Table 5 Gene Ontology enrichment results for genes associated with *P. gingivalis* detection.

Supplementary Table 6 Neuroanatomical enrichment results for genes associated with *P. gingivalis* detection.

Supplementary Table 7 Nervous system cell-type enrichment results for genes associated with *P. gingivalis* detection.

Supplementary Table 8 Neuroanatomical enrichment results for the SRP-dependent cotranslational protein targeting to membrane genes.

Supplementary Table 9 Nervous system cell-type enrichment results for the SRP-dependent cotranslational protein targeting to membrane genes.

Supplementary Figure 1 Gingipain digestion of 70S ribosomes. Fifteen microliter reactions containing 2.5 ug of 70S ribosome and either 120 nM of recombinant Lys-gingipain (rKgp), Arg-gingipains (rRgpA and rRgpB), or about 206 – 412 µM (one unit in a 15 µL reaction volume) of Caspase-3 were incubated at 30°C for 1 h or 3 h. After incubation, results were visualized using SDS PAGE &Coomassie brilliant blue staining. The Precision Plus Protein WesternC Dual Color ladder was used for band sizing. The asterisk (*) to the left of the ribosome only control indicates a faint band present at about 60 kDa. This faint band appeared to be digested by Kgp and RgpB after 1 and 3 hours of incubation, as indicated by carets (^) to the left of the Kgp and RgpB.

## Abbreviations

AD, Alzheimer’s disease; AmG, amygdala; ApoE, apolipoprotein E; AUC, area under the receiver operating characteristic curve; BF, basal forebrain; BG, basal ganglia; CHAT, choline o-acetyltransferase; EP, epithalamus; ER, endoplasmic reticulum; FL, frontal lobe; GO, Gene Ontology; HiF, hippocampal formation; Hy, hypothalamus; ITM2B, integral membrane protein 2B; Kgp, Lys-gingipain; MAPT, microtubule-associated protein tau; MAZ, Myc associated zinc finger protein; MES, mesencephalon; MET, metencephalon; MY, myelencephalon; NOS, nitric oxide synthase; OL, occipital lobe; P. gingivalis, Porphyromonas gingivalis; PL, parietal lobe; RgpA, gingipain R1; RgpB, gingipain R2; RPL41, ribosomal protein L41; SAF-1, serum amyloid A-activating factor-1; SRP, signal recognition particle; SRP9, signal recognition particle 9; TH, thalamus; TL, temporal lobe.
