# Supplementary material for: Characterization of Human Genes Modulated by Porphyromonas gingivalis Highlights the Ribosome, Hypothalamus, and Cholinergic Neurons
Source: Front Immunol. 2021 Jun 14;12:646259. doi: 10.3389/fimmu.2021.646259 (PMC8236716; doi:10.3389/fimmu.2021.646259)
Supplement: Supplementary Figure 1 — Gingipain digestion of 70S ribosomes. Fifteen microliter reactions containing 2.5 ug of 70S ribosome and either 120 nM of recombinant Lys-gingipain (rKgp), Arg-gingipains (rRgpA and rRgpB), or about 206 – 412 µM (one unit in a 15 µL reaction volume) of Caspase-3 were incubated at 30°C for 1 h or 3 h. After incubation, results were visualized using SDS PAGE &Coomassie brilliant blue staining. The Precision Plus Protein WesternC Dual Color ladder was used for band sizing. The asterisk (*) to the left of the ribosome only control indicates a faint band present at about 60 kDa. This faint band appeared to be digested by Kgp and RgpB after 1 and 3 hours of incubation, as indicated by carets (^) to the left of the Kgp and RgpB. [file Image_1.pdf]

Supplemental Figure 1

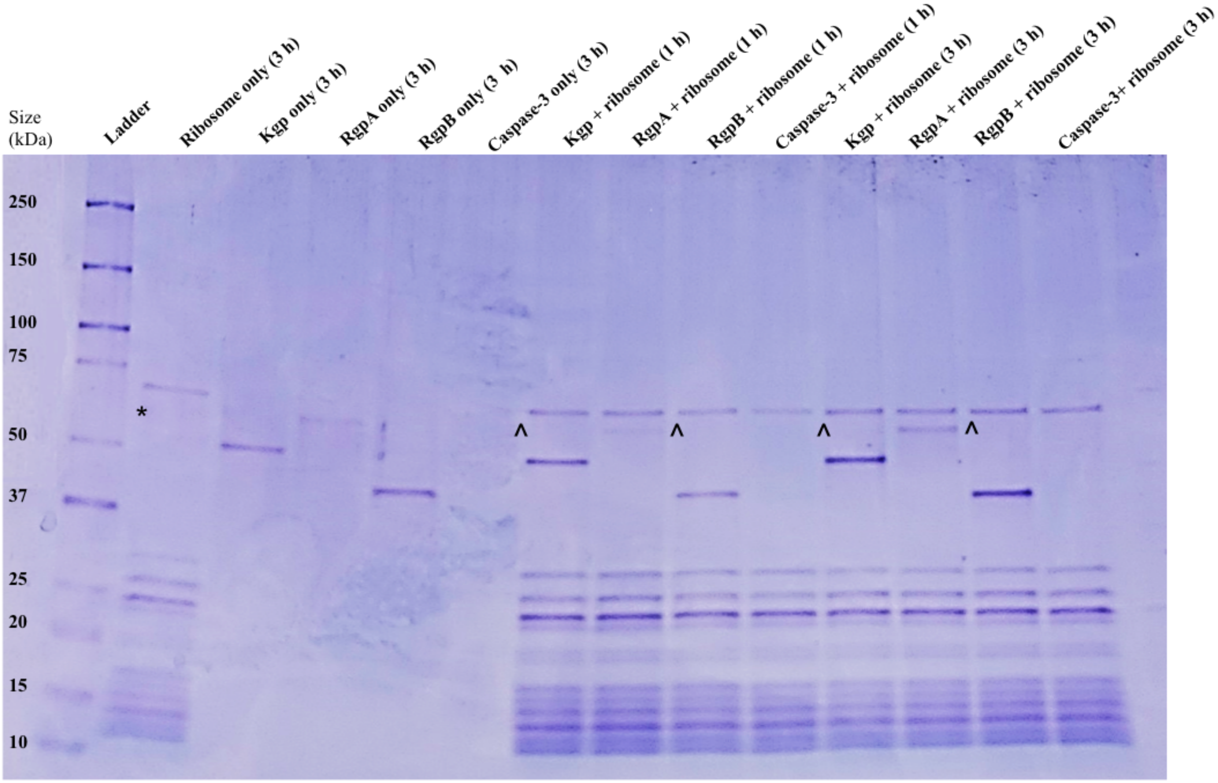

Supplemental Figure 1: Gingipain digestion of 70S ribosomes. Fifteen microliter reactions containing 2.5 ug of 70S ribosome and either 120 nM of recombinant Lys-gingipain (rKgp), Arg-gingipains (rRgpA and rRgpB), or about 206 – 412 µM (one unit in a 15 µL reaction volume) of Caspase-3 were incubated at 30°C for 1 h or 3 h. After incubation, results were visualized using SDS PAGE & Coomassie brilliant blue staining. The Precision Plus Protein WesternC Dual Color ladder was used for band sizing. The asterisk (*) to the left of the ribosome only control indicates a faint band present at about 60 kDa. This faint band appeared to be digested by Kgp and RgpB after 1 and 3 hours of incubation, as indicated by carets (^) to the left of the Kgp and RgpB.
